# Extract from Observations upon Anæsthesia of the Cornea and Radiating Fibres of the Iris Occurring without Intra-Ocular Tension

**Published:** 1867-05

**Authors:** Jos. S. Hildreth

**Affiliations:** Opthalmic and Aural Surgeon to Cook County Hospital, Chicago, Ill.


### EXTRACT FROM OBSERVATIONS UPON ANÆSTHESIA OF THE CORNEA AND RADIATING FIBRES OF THE IRIS OCCURRING WITHOUT INTRA-OCULAR TENSION.

By JOS. S. HILDRETH, M.D., Opthalmic and Aural Surgeon to Cook County Hospital, Chicago, Ill.

By anæsthesia of the cornea I here refer to that diminution of its sensitiveness to touch which may exist without apparent organic disturbance of the part.

By anæsthesia of the radiating fibres of the iris, to a condition of this membrane which, without mechanical complication or apparent organic change, prevents the pupil from properly responding to the influence of atropia or light.

The degree of anæsthesia may be ascertained by gently touching the cornea obliquely with the point of a small, soft camel's hair pencil, previously wet and stripped quite dry; or the point of a small twisted roll of unsized paper slightly moistened.

This should not be repeated more than once or twice during a minute, lest a degree of toleration be established which would interfere with correct appreciation.

The iris is to be tested by using a given number of drops of atropia in solution of definite strength.* But its dilatability does not always correspond to the state of the cornea, being in some cases greater with the same degree of anæsthesia than in others; yet, the amount of atropia and time required for dilatation are valuable diagnostic guides. The influence of light should also be noted.

These pathological conditions may exist from a slight to a very marked degree, and doubtless are caused by a certain abnormal state of the ciliary apparatus, which may result from

* Four grains to the ounce.

intra-orbital, cranial, or spinal causes; and possibly, in some cases, be idiopathic. It can also be temporary, as it were, spasmodic, or become permanent.

The influence exerted by this affection upon other diseases, especially panniform and ulcerated keratitis, infiltration and sloughing of the cornea in purulent ophthalmia, panus, and some forms of atrophy of the globe, engages particular attention.

When slight, of recent origin, or spasmodic, it may, in some cases, be relieved by medical means only; but, if existing to a high degree, or of long standing, division of the ciliary ring becomes necessary.

The following clinical facts are taken from the records of the United States Eye and Ear Hospital. All were operated upon without ether or chloroform.

Furrow, æt. 29. April 13, 1865. Left eye. Cornea considerably anæsthetized, panniform, and centrally ulcerated; pupil only slightly influenced by atropia. No apparent change of iris, *no tension of globe;* acute pain in and about the orbit.

Division of ciliary ring.*

The corneal anæsthesia was at once relieved, and the pupil became dilated in thirty minutes, though no atropia had been used for more than twenty-four hours. Pain speedily abated.

June 19. From the date of the operation there had been no return of pain; the pupil readily responded to atropia, but was not fully susceptible to light. The reflex movements were more marked from than to this side. No corneal anæsthesia remained; its panniform condition had greatly improved, and ulceration healed. Periphery of retina apparently unimpaired.

General and local medical treatment previous to the operation had failed to afford relief.

Crippen, æt. 20. April 13, 1865. Right eye. Cornea panniform, ulcerated, and considerably anæsthetized. Pupil slightly influenced by atropia. No other affection of iris; *no tension of globe;* severe pain in and about the orbit.

Ciliary ring divided as in preceding case.

Corneal anæsthesia immediately disappeared, and the pupil dilated in ten minutes, though no atropia had been used for twenty-four hours. Pain in orbit at once relieved, and from surrounding parts in a few days.

June 19. The corneal anæsthesia had not returned; the

* The point of operating was in all of these cases between the external and inferior recti, unless repeated, when it was between the inferior and external.

pupil dilated easily, and responded to light. Ulceration had healed, and panniform condition was improving. No return of pain. Periphery of retina unaffected, and reflex movements of irides equal.

Medical treatment had failed to afford relief.

Lamont, æt. 49. April 26, 1865. Left eye. Pain of varying intensity, radiating to corresponding supra-orbital and coronal regions. Cornea anæsthetized, and iris only feebly influenced by atropia. *No tenison of globe.*

General and local medical treatment, with frequent paracentesis of the anterior chamber, had failed to materially improve these conditions.

The ciliary muscle was divided obliquely backward from its corneal attachment, about four and a-half or five millimetres.

Corneal anæsthesia was at once largely diminished, and the pupil dilated in about twenty minutes. Pain in the orbit relieved within a few minutes, and in coronal and supra-orbital regions in forty-eight hours. No atropia had been used within twenty-four hours of the operation.

In the course of ten days, the pupil, having been allowed to contract again, was found beyond the influence of atropia. The corneal anæsthesia was increasing and pain returning.

May 13. The ciliary muscle was again divided from its anterior insertion obliquely backward about six and a-half or seven millimetres.

The immediate effect was, as before, to remove the corneal anæsthesia, and to allow the pupil to dilate in about twenty minutes, from the effects of atropia that had been used forty-eight hours previously, as none had been employed since that time.

October 16. The corneal anæsthesia had not returned, the pupil was perfectly free, and responded to the action of atropia and light. Its contraction by reflex action was not so perfect as that of the opposite side.

Ulceration of the cornea had healed.

Peripheric vision, with dilated pupil, was good.

The imperfect results following the first operation are attributed to imperfect division of the affected parts of the ciliary ring. But it is probable all of its effects would not have been lost, as they were, had the use of atropia properly been kept up.

Hughes, æt. 23. Right cornea anæsthetized, and the pupil slightly affected by atropia.

Medical treatment, with repeated and systematic paracentesis of the anterior chamber, slightly influenced these conditions.

May 11. The pupil contracted; was not affected by atropia, and the cornea was still anæsthetized. No other apparent affection of the iris, and *no tenison of the globe.*

Division of the ciliary ring, with the immediate effect of removing the corneal anæsthesia, and causing the pupil speedily to dilate, though no atropia had been used for twenty-four hours.

June 19. The pupil has been dilatable since the operation, and anæsthesia of the cornea has not returned.

Peripheric vision of this eye apparently good, and reflex movements of irides are equal.

Pemberton, æt. 29. March 18. The cornea of the right eye anæsthetized, and the pupil feebly influenced by atropia; considerable pain in and about the orbit. No intra-ocular tenison.

The ciliary ring was divided, which immediately removed the anæsthesia of the cornea and caused the pupil to speedily dilate.

No atropia had been used for twenty-four hours.

June 17. The corneal anæsthesia has not returned; the pupil dilates readily and responds to light. Periphery of retina unaffected, and reflex movements of irides equal.

The above cases represent the pathological conditions under observation, and show the results of division of the ciliary ring upon them after general and local medical treatment, aided in a number of them by frequent and systematic paracentesis of the cornea, had failed.

In no instance did evacuation of the aqueous humor affect the anæsthetized condition of the cornea or allow the pupil to dilate, though atropia was at the time freely used.

The influence of these conditions upon some forms of keratic disease is also above made apparent.

In the following case partial atrophy of the globe and corneal anæsthesia were both relieved by division of the ciliary ring after iridectomy had failed.

Hamilton, æt. 20. Left eye. Large leucoma, with anterior synechia; remainder of cornea nebulous and anæsthetized. Slight atrophy of the globe. Iridectomy, September 15, 1865.

October 11. Anæsthesia of cornea remains, and atrophy of globe unchanged.

Division of the ciliary muscle caused the corneal anæsthesia to disappear within forty-eight hours.

November 6. Globe is firm, and cornea normally sensitive.

The form of corneal insensibility under observation is usually uncomplicated; but it may co-exist with intra-ocular tenison, as in the following instance:

Johnson, æt. 25. Left eye. Staphyloma of choroid; iris dark and disorganized; pupil contracted and bound by posterior synechia; cornea anæsthetized; intra-ocular tenison well-marked.

October 30. Iridectomy followed in fifteen minutes by division of the ciliary muscle.

After the removal of one-sixth of the iris from its pupillary to the ciliary margin, the cornea remained anæsthetized as before, but was immediately relieved by the second operation.

In two other cases the pupil quite undilatable, yielded upon administering ether to a high grade of relaxation.

Dilatation became quite full in one, was easily maintained by atropia, and permanent relief followed. Only partial in the other; on discontinuing the drug, the pupil contracted and refused to dilate as before.

Several times I have observed this condition of the iris to be carried by reflex action to the opposite side. So that, upon relieving the eye most affected, it gradually disappeared from the other.

In some instances a certain grade of anæsthesia has been modified by well dilating the pupil, which accounts for the favorable influence of this remedy in keratic affections.

Von Graefe remarked, in 1859, that if the pupil readily dilates by atropia, there would be less liability to suppuration after extraction of cataract, or words to that effect.

That condition producing the corneal anæsthesia, specially described in this paper, undoubtedly often has been instrumental in producing unfavorable results in this operation, and it should be removed before flap extraction.

The early diagnosis of that condition represented by corneal anæsthesia and iritic insusceptibility to atropia, is of the greatest importance in purulent ophthalmia, as it is frequently followed by infiltration and consequent sloughing of the cornea or ulceration from the epithelial surface without previous infiltration.

If the narrow and dense band of conjunctiva immediately surrounding the cornea remains intact, infiltration of the sub-epithelial structures will precede sloughing; but if this conjunctival band early become infiltrated or disorganized, sloughing from the surface soon follows, though the parts beneath may not be infiltrated.

I would operate in all such cases when anæsthesia of the cornea could not be relieved by atropia, and especially when the pupil had become undiltable, and then *maintain the effects of the operation by the use of atropia.*

As stated in a previous paper, this operation should not wholly be relied upon in such cases. Other means must also be used to control inflammatory action, the purulent discharge, and the chemosis. By its proper and timely use, the cornea can be saved when all other known means *combined* would inevitably fail.

The successful treatment of panus by syndectomy and purulent inoculation involves one of the causes of its original production—imperfect nutrition of the deep tissues of the cornea.

From results already recorded, and experiments in progress, it is evident pannus may yet be successfully treated without resort to purulent inoculation.

As this paper is intended only to report certain clinical facts, theoretical deductions, as far as practicable, have been omitted.—*Transactions of the Am. Ophthalmological Society.*

